# 

**Published:** 1883-04

**Authors:** 


CONTENTS.

| | Page. | | Page. |
|---|---|---|---|
| Colds | 85 | Weak Eyes | 93 |
| Appropriate Foods | 86 | Slaves of the Cooking Stoves | 94 |
| Salted Meats | 87 | Dangers of the Cold Bath | 95 |
| Rice and Beans | 88 | Cataract of the Eye | 96 |
| Quacks and their Methods | 89 | Miscellaneous Items | 97 |
| Driven Wells | 90 | Food Adulterations | 99 |
| Constipation | 91 | Bill of Fare | 101 |
| Feet and Shoes | 93 | Popular Science | 102 |

